# Subglottal and bronchial wall involvement in granulomatosis with polyangiitis identified on CT-PET with initial misdiagnosis of metastatic cancer

**DOI:** 10.1093/rap/rkad106

**Published:** 2023-12-01

**Authors:** Azeem Ahmed, Shivani Gor, Khin Yein, Shabeena Zeb

**Affiliations:** Rheumatology Department, Great Western Hospital, Swindon, UK; Rheumatology Department, Great Western Hospital, Swindon, UK; Rheumatology Department, Great Western Hospital, Swindon, UK; Rheumatology Department, Great Western Hospital, Swindon, UK

A 66-year-old female non-smoker presented with dyspnoea, cough and fever. She had a history of breast cancer managed with mastectomy. A right-sided hilum shadowing on chest X-ray (Fig. 1A) prompted a CT scan. This revealed a right middle lobe mass and multiple lytic lesions in the spine suggestive of bony metastases (Fig. 1B, C). CT-PET highlighted thickening of the trachea from the level of the suprasternal notch extending into both main bronchi (Fig. 1D–F). Subpleural apical consolidation along the lateral pleural surface (Fig. 1E) and wedge-shaped consolidation in the middle lobe (Fig. 1G) were [^18^F]fluorodeoxyglucose avid. Diffuse homogeneous uptake in the spine was reactive and not metastases (Fig 1D, E). Biopsy revealed necrotizing granulomatous inflammation. cANCA was positive, with a PR3 level of 15 IU/ml, confirming granulomatosis with polyangiitis. She started prednisolone and CYC and switched to AZA for maintenance.

**Figure 1.**
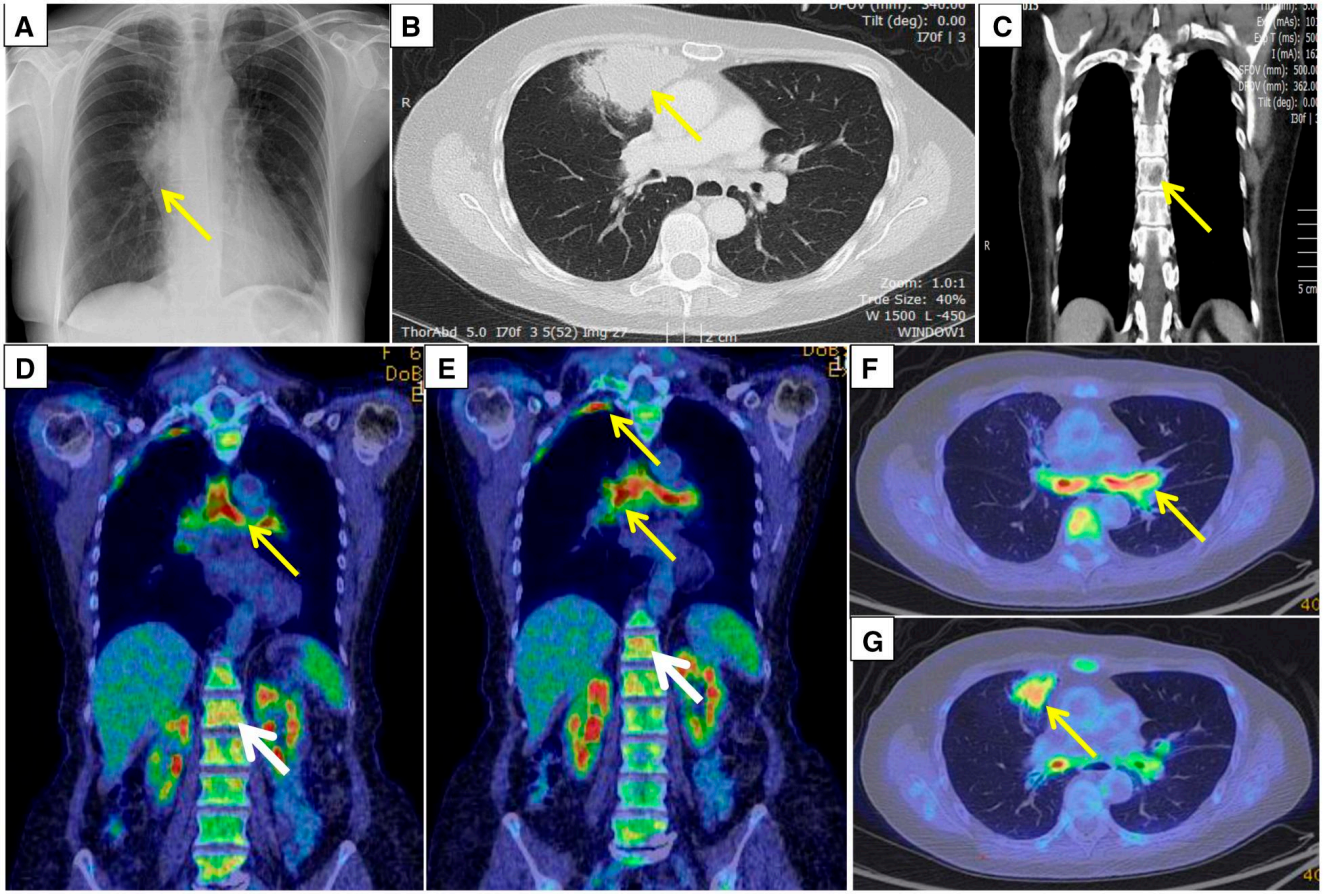
Chest X-ray, CT thorax and CT-PET imaging. (**A**) Chest X-ray, showing new right hila shadowing. (**B**) CT thorax image, showing right middle lobe mass with air bronchogram. (**C**) Multiple lytic lesions in the spine, suggesting bony metastases. (**D–F**) [^18^F]Fluorodeoxyglucose-avid trachea and bronchi and inferior pleural surface (yellow arrows). (**D**, **E**) Reactive homogeneous increase in marrow uptake (white arrows). (**G**) Wedge-shaped consolidation in the right middle lobe

Two years later, she developed stridor. ENT examination demonstrated active inflammation at the subglottis. She received rituximab for relapsing disease, with improvement in her airways and with normal levels of CRP (2.7 mg/l) and PR3 (0.8 IU/ml).

Our case highlights the benefits of CT-PET to identify subglottal and bronchial wall involvement in granulomatosis with polyangiitis. Subglottal involvement can occur in 16–23% of patients [1]. Although subglottal involvement does not usually respond to immunosuppression, rituximab can be useful in the early stages, when significant narrowing has not been established [2].

## Data Availability

No new data were generated in support of this manuscript.
